# Analysis of the Possibility to Detect Road Vehicles via Bluetooth Technology

**DOI:** 10.3390/s21217281

**Published:** 2021-11-01

**Authors:** Răzvan Andrei Gheorghiu, Valentin Iordache, Angel Ciprian Cormoș

**Affiliations:** Transport Faculty, Politehnica University of Bucharest, 060042 Bucharest, Romania; valentin.iordache@upb.ro (V.I.); angel.cormos@upb.ro (A.C.C.)

**Keywords:** Bluetooth detection, RADAR, road traffic, vehicle detection, origin–destination matrix

## Abstract

As road traffic networks become more congested and information systems are implemented to manage traffic flows, real-time data gathering becomes increasingly important. Classic detectors are placed in one point of the network and are able to provide information only from that area. As useful as this is, it lacks the big picture of the routes the vehicles usually travel. There are applications developed to help individuals make their way into the road network, but these are no solutions that deal with the cause of traffic; rather, they counteract the effects. It becomes obvious that a proper management system, with knowledge of all the relevant aspects will better serve all travelers. The detection solution proposed in this paper is based on Bluetooth detectors. This system is able to match detected devices in the road network, filter the results, and generate a vehicle count that is proved to follow RADAR detection results.

## 1. Introduction

Road traffic is a critical discussion in all countries. Talking about basic regulations, safety for different participants, or advanced traffic systems (maybe including even artificial intelligence), the critical point is the same: knowing the current status and determining the problems. This can be done manually, via observations or manual traffic count, but this has the downside of data collection only in specific points and in specific time intervals. The alternative is represented by automated traffic counts, and here we find many solutions and technologies: from inductive loops placed under the pavement and detecting metal parts in the vehicles passing by (a disadvantage for new, composite materials that cannot be detected this way), to RADAR or infrared detectors, a mature and reliable technology, to video cameras that can extract many features (much more than any other sensor) but with the downside of high costs for equipment, detection software, and so on.

**Figure 1:**
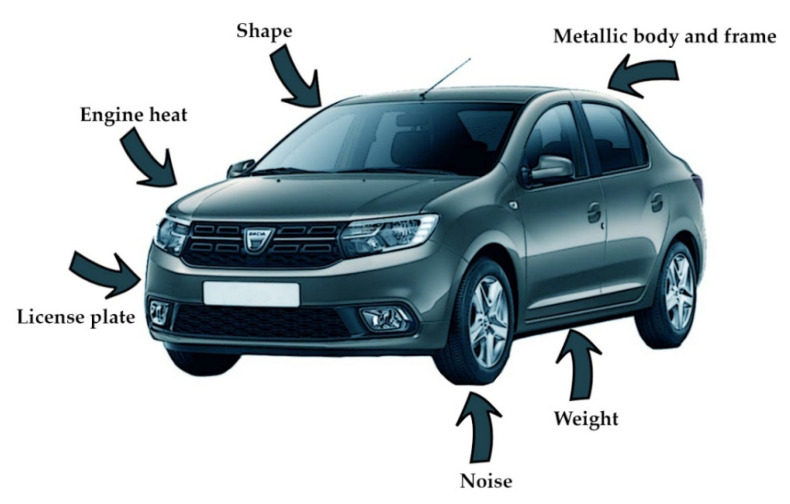
Vehicle parts considered for different types of traffic sensors.

Vehicle detectors technologies are constantly improving, and new types are emerging, based on the presence of new on-board systems and devices. For many years, detection was based only on the vehicle’s physical characteristics or how it is affecting the environment (Figure 1): pneumatic road tubes and weigh in motion systems use the weight of the vehicle as information for its presence, magnetic sensors and inductive loop detectors sense the vehicle because it has a metallic body and frame, microphones are used to capture its noise signature, video image processing extracts the shape of it or read the license plate number, infrared sensors are sense the heat generated by the engine, and so on.

These solutions are usually based on expensive sensors. Considering that the traffic increase problems are spreading to smaller cities that usually do not have big budgets that can be allocated for traffic management systems, it seems that with current used technologies there is no solution in those locations. Therefore, the search for less expensive solutions is required. In addition, we may notice the advancement of navigation applications that do not use an exact number of vehicles, but follow the users’ behavior in the road network and are able to provide enough useful information based only on speeds and travel times.

**Figure 2:**
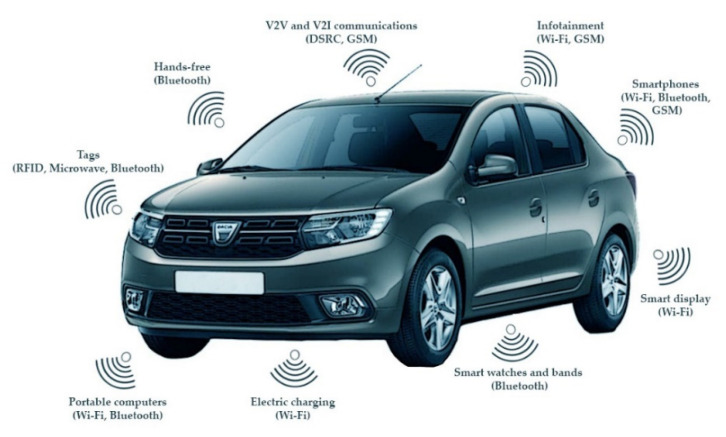
On-board communication technologies.

Newer and evolving detection systems are currently considered to gather information about vehicles’ presence and characteristics through on-board wireless communication systems. Different technologies are being implemented to better support the safety and security of vehicles, to provide services, and to increase the comfort of the driver and passengers, to make driving autonomous, or to access vehicle diagnostics (Figure 2). Dedicated technologies like DSRC (dedicated short-range communications) are used to create wireless links between the vehicle and the road infrastructure or other vehicles (V2X—vehicle to everything, regardless the communication technology), as well as widely available commercial technologies like Bluetooth, Wi-Fi, or cellular to create personal networks or hotspots inside the vehicle, or to gain access to the Internet. These new solutions can complement older ones to provide better detection accuracy and, in some cases, more relevant data.

Among the mentioned technologies, Bluetooth seems to be more appropriate for use in vehicle detection, as it is highly used in individual portable devices to communicate in small area personal networks, and the number of devices per person continues to increase, from 2.4 in 2018 to 3.6 by 2023 [1].

Bluetooth allows a device to discover devices in their vicinity and read their MAC address, which are unique for every device, without establishing a connection, thus offering a method of detecting and individualizing vehicles. Furthermore, filtering of the detected Bluetooth devices can be done by using the dedicated inquiry access code (DIAC), a code used in the device discovery process that identifies the device class (computer, phone, audio/video, wearable, etc.) and the service class (audio, telephony, positioning, networking, etc.). Last but not least, a great advantage against other types of detectors is that the cost is relatively low for a Bluetooth scanner, and maintenance is hardly needed.

The scope of this paper is to determine whether Bluetooth (as a cheap detector) is able to follow a real traffic pattern and, by providing information about speeds, travel times, and travel patterns via origin–destination matrices, whether it can be an alternative solution to expensive traffic monitoring systems.

The paper is organized as follows. Section 2 presents a brief review of the related work regarding key issues of Bluetooth detector systems, key applications and traffic information that can be obtained, performance of research proposals, and examples of commercial systems. Section 3 describes the used platforms and the use cases. The analysis of the traffic related information is performed in Section 4. Finally, Section 5 is devoted to the Conclusions.

## 2. Related Work

A key issue, when dealing with Bluetooth devices in road traffic, is the detection rate, which should be as high as possible so that the information gathered can be considered reliable. On this matter, a study published in 2019 [2] that covered the years 2010 to 2016, showed that the detection rate was of about 25%. Also, in [3], the authors compared Bluetooth detections with the real number of vehicles transiting an intersection, and determined with the help of a video camera, a detection rate of about 30%. Although there are other studies showing smaller detection rates [2,4,5,6,7], it is important to take into consideration the fact that technological evolution and improvements in detection methods can lead to increased performance.

The detector scans for Bluetooth devices, encrypts the data for privacy reasons, and sends it to the centralized server for processing. Based on the information gathered, the system can identify MAC address matches between pairs of detectors and apply a time stamp, and thus calculate the travel time between the two locations, considering that the MAC address of a device is unique. This key application of detecting vehicles by using multiple Bluetooth detectors is studied in many papers [4,8,9,10,11,12]. In particular, the authors of [13] provided a travel time estimation based exclusively on vehicles, studying issues like intrinsic errors, multiple detections, and outliers. Their methodology provided solid estimations, 89% of them having an error lower than 10%, for a 95% confidence level. Travel time values were also used in [9,14] for an automatic incident detection with good results if the incident or its effects are directly located within the coverage area of the Bluetooth sensor. An extensive study is presented in [15], demonstrating the possibility of deriving performance measures such as travel time and vehicle speed to enhance traffic management using Bluetooth.

Vehicle origin–destination data can use Bluetooth detection as a solution. In [10] the authors compared Bluetooth data with ANPR and video origin–destination matrices with favorable results, although the correction and expansion methodologies would need improvement. The authors of [16] proposes a more complex matrix, the origin–destination link, and demonstrated the results of its implementation for the city of Brisbane, where over 60,000 Bluetooth detectors have been installed.

The short- and long-term road traffic situation is estimated in [17], based on a combination of the number of detected Bluetooth devices and the weather condition data obtained from distributed sensors. The results showed that the smallest average relative error between the estimated number of cars and the actual traffic was less than 10%. In [18], the authors performed a traffic variability and dynamics analysis using a multi-sensor Bluetooth network for traffic volume monitoring, concluding that the long-term research indicated only minor variation in the number of vehicle detections and relatively stable traffic volume.

The authors of [19] proposed a vehicle classification approach by using Bluetooth beacons for road traffic monitoring. High accuracy was achieved when introducing a dedicated ensemble of random forest classifiers with majority voting. A method of filtering data and classifying road users is presented in [17], splitting them into three categories: (1) pedestrians, cyclists, or a people in stationary vehicles; (2) cars or trucks; or (3) buses.

Data from other sources were often fused or compared with data obtained by Bluetooth detectors. In [8] the authors integrated data from Bluetooth scanners with inductive loop data to obtain a better estimation of the travel time and detailed route of a vehicle on a motorway, and the proposed model provided improvements in accuracy of over 10%. The detection rate was compared in [2,9], concluding that about 33% and 25%, respectively, of all vehicles were detected, considering the average daily traffic data obtained from inductive loops. Loop detectors and microwave sensors were used as ground truth in [5], with the authors concluding that the maximum average detection rate was of 8.13%.

The authors of [20] concluded that Bluetooth estimates of travel speed tend to be very close to measurements taken with GPS probe vehicles, and the fused estimate from loop detector and Bluetooth data will usually have equal or better accuracy. Floating car data (FCD) from 152 GPS-trace equipped vehicles over a period of 2 years was used as ground truth in [11], with the results showing that the variance of Bluetooth-based travel time estimates was significantly higher than the FCD estimates, possibly due to the larger detection zone and the low penetration rate. Probe vehicles were also used in [4,12] along with video images that were recorded during the Bluetooth data collection, and it was found that the Bluetooth system was able to report slightly higher travel times, but with results close to those of the ground truth method. In [21], crowdsourced GPS data from the TomTom company was used as ground truth to evaluate the accuracy of Bluetooth average travel time, and the results showed that the differences were systematic and that performing a calibration could be a solution for correcting inaccurate travel times. Data from another private sector supplier, HERE, was used in [22], with the author demonstrating that Bluetooth data has better accuracy and reliability.

Traffic data were measured using a Bluetooth detector and a RADAR by the authors of [23]. The occupancy of a point on a road was determined using both technologies, then by using Pearson’s and Spearman’s coefficients it was determined that there is a positive strong correlation between the two different measurement results. A RADAR detector was also used as ground truth in [5], concluding that the maximum average detection rate was 5.39%, and in [24], with values between 10% and 12.19%. A passive detection system was used in [25] along with a machine learning algorithm to obtain more accurate traffic flow estimates from the Bluetooth data. Pneumatic tube sensors were used in the same locations as the detectors to determine the real travel speed, and it was determined that the Bluetooth estimated median values had a greater variance, but were comparable.

Without regard to the application, several issues affect proper vehicle detection. The speed of the vehicle is important. Although there are detectors that are proven to be able to detect vehicles at high speeds [9], it has been showed that detection probability is higher if it takes longer for the device to cross the area of the detector [26], and that means slower speeds. The same study states that the travel direction is significant: a vehicle is more likely to be detected when it is approaching the scanner. On the other hand, slower speeds may lead to another problem: The appearance of multiple detections, representing the number of detections for a unique MAC address during the passage of the vehicle through the detector area, which will lead to erroneous detection rates, up to 25% of all detections in [9], or 48% in [27]. For travel time estimation usually the first detection is considered [8], and for calculation of the dwell time (the time spent by the vehicle in the detector area) the first and the last detection [23]. Dwell time in intersections was also considered important in [28] to be able to infer the most likely route used by a vehicle between two successive Bluetooth detections. 

A more complicated issue is the detection of multiple devices on board a single vehicle [10], which may lead to erroneous vehicle count. Solutions are presented in [9] and the author’s estimation was that up to 15% of all detections are subject to this issue. The authors of [21] proposed several scenarios with different solutions to the multiple detection problem. Also, although MAC addresses are expected to be unique, it appears that sometimes the same address seems to be shared amongst different vehicles, possible because of MAC cloning situations or because those vehicles are frequent users of the road network [29]. An algorithm for detecting multiple Bluetooth devices is presented in [30].

Although there are concerns that Bluetooth alone is not a proper technology for travel time measurements [11,31], many traffic-related technology companies are considering developing and commercializing Bluetooth detectors. BlipTrack [32] is used in airports and cities to gather data about travel time, origin and destination, traffic flow and queuing. The BlipTrack algorithms filter unwanted detections like parked cars, cars changing direction, trains or busses passing by, or even bicycles and pedestrians. The city of Aarhus in Denmark conducted eight months of testing on this system and the results showed that Bluetooth detectors could offer the same information as alternative and more expensive solutions. SensID, a detector provided by Sensys [33], can measure travel times, perform congestion mapping or origin/destination analysis, and support Wi-Fi detections. Another type of detector incorporating Bluetooth and Wi-Fi is called DeepBlue D-Model [34], and it can be used to determine travel times, incidents in traffic and OD matrices, allowing vehicle detection for up to 12 lanes of traffic. The Siemens Sapphire Journey Time Measurement System [35] offers an extended detection range of up to 100 m for traffic environments and can be used to measure travel times, being able to automatically filter any static devices. The eScan Bluetooth scanner [36] also uses a Class 1 Bluetooth module for an extended detection range and provides highly accurate journey time samples. Another type of combined detector, Bluetooth and Wi-Fi, is TrafficXHub [37] which provides superior MAC address detection and matching.

**Figure 3:**
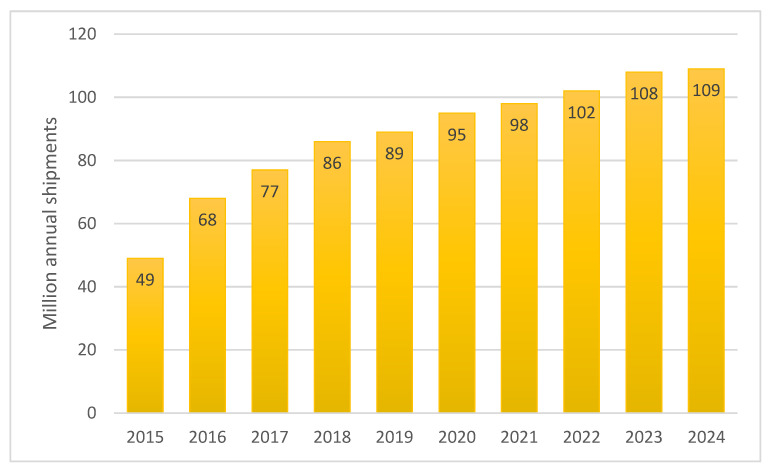
Bluetooth automotive device shipments (data available at [38]).

In summary, although the results of the above analyzed studies show that Bluetooth can be considered a promising vehicle detection solution dedicated to indicating the state of traffic flow (see potential applications in Table 1) almost all researchers believe that further studies and tests are needed. Bluetooth detectors are very popular due to their cost-effectiveness and ease of installation, and they will become a strong alternative to traditional traffic measurements technologies if their detection rate continues to increase. This improvement will probably be based on several factors, including:

- The number of Bluetooth devices per person is expected to grow.
- 87% of all new vehicles currently come standard with Bluetooth technology, and by 2024, two thirds of all cars on the road will include it [38].
- Bluetooth automotive device shipments will continue to grow (Figure 3).
- Technology evolution such as Bluetooth 5 can increase the communication range by four times compared to version 4 [39], which will help increase the detection rate of vehicles, as they will spend more time in the area of a detector, and Bluetooth 5.1 added a new feature called “direction finding” [40], which offers two different methods for determining the angle from which a Bluetooth signal is transmitted with a high degree of accuracy, creating the potential to allow scanning of separate road lanes, eliminating the possibility of detect pedestrians, or helping detect multiple devices aboard one single vehicle.

Considering the factors presented above, it is important to assess the advances of this detection technology, especially in real and commercial implementations, along with improvements to detection rates, in various geographical locations and in networks with different traffic characteristics. The following work will make use of two different platforms and several case studies in which Bluetooth detectors are used to gather road traffic data, analyzing it to point out the usefulness and effectiveness of these new implementations. For one of them, RADAR data is available, and it will serve as ground truth to estimate the detection rate.

## 3. Bluetooth Detection Systems Description

### 3.1. Used Platforms

Bluetooth detection solutions can be implemented using dedicated platforms that will collect data from detectors, filter and analyze it, and generate reports useful for the users. In this paper, two platforms that manage Bluetooth detectors and provide information based on them were used: one of them is the Virtual Control Center (VCC) [41], provided by TrafficNow, Spain, and the Analytiqum mobility platform for smart systems (AMP4SS) [42], provided by Analytiqum Innovative Solutions, Romania.

**Figure 4:**
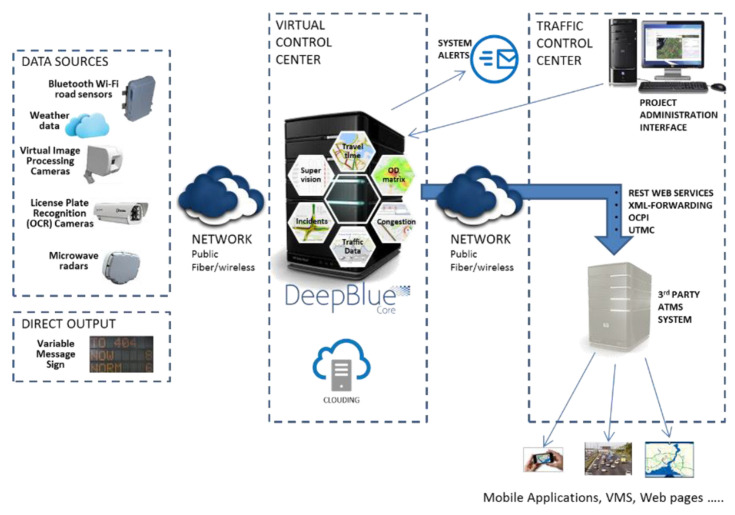
VCC platform architecture [41].

Figure 4 presents the general architecture of such data collecting platforms and their intended usage.

For the implementation of the systems that were used to extract data, Bluetooth detectors were installed in the road network, namely in each relevant junction (at the boundary and inside the target area). Junctions that have very low traffic (therefore a low estimated detection rate), or in which vehicles mainly travel forward (very few turns) were not considered. Each detector is able to identify the MAC addresses of the devices in its area, and sends them, along with a timestamp, to a central server (a cloud solution) for processing. At the central level, dedicated software matched MAC identifiers from adjacent sensors, thus identifying objects that travel through the road network. Afterwards, a filtering procedure was applied to eliminate improper data (like objects travelling too slow compared to the average speed). Also, a clustering method was implemented, to group devices travelling in the exact pattern (that can be, for example, travelers inside a public transport vehicle). For the user interface, several Web platforms were developed and connected to the database to represent the information on a map or in tabular form.

Considering how Bluetooth works, it is important to emphasize from the beginning the expectations and tests that were performed: as this technology is not present in all vehicles, we cannot expect to obtain an accurate vehicle count. However, it is important to test if the traffic pattern detected with this solution follows the real one. In this case, the traffic characteristics determined for a relevant sample data will be true for the whole traffic flow. The data are related mainly to speeds and travel times but, due to the displacement of the sensors in the road network, also origin–destination data can be obtained.

**Figure 5:**
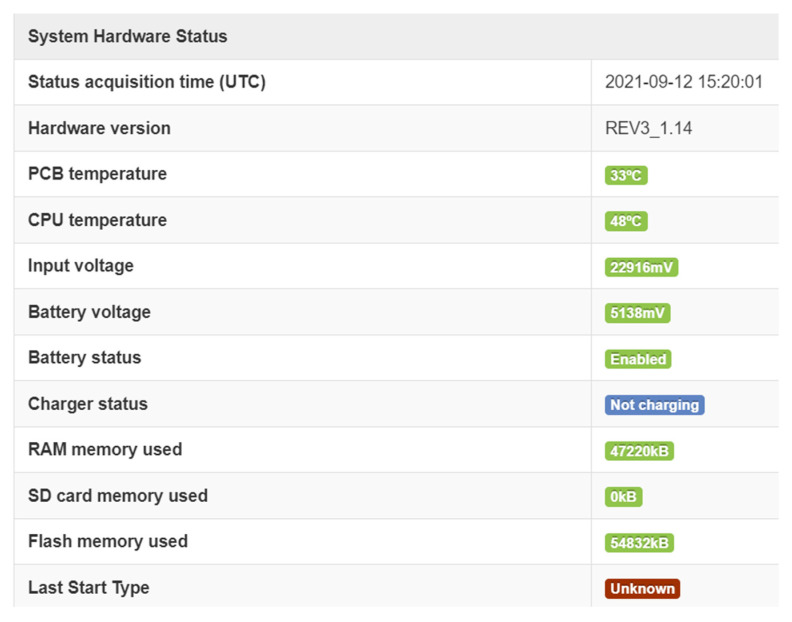
Example of detector monitor information.

Using this support, Bluetooth detection systems were implemented/proposed for testing in several cities. We have chosen some examples to show the detectors infrastructure that is required in A Coruña (Spain), Vigo (Spain), or Alba Iulia (Romania). A short description of each project and a figure illustrating the area covered by the system are presented below. The sensors used are based on an ARM 9 processor, with 128 MB RAM Flash, and a Bluetooth detection internal 15dBi antenna. They were all continuously monitored to determine if the system performed at its best (Figure 5). Temperatures, both for PCB and CPU were checked, and the battery was tested to ensure proper functioning, and the memory was monitored (both volatile and non-volatile).

### 3.2. Use Cases

**Figure 6:**
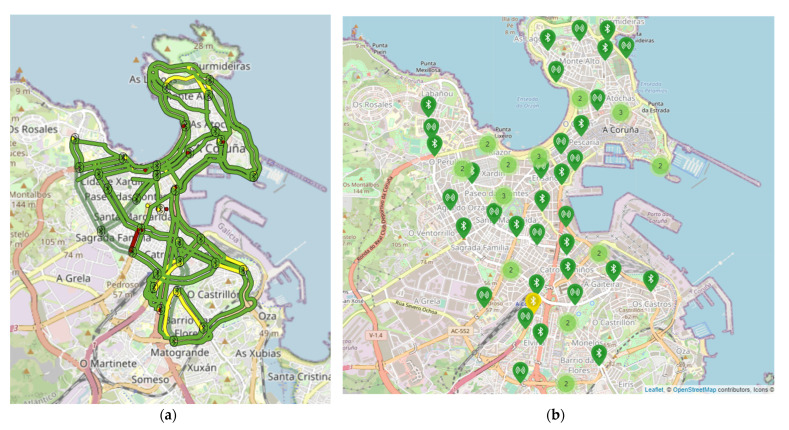
A Coruña implementation and detector placement: (**a**) from VCC platform; (**b**) details from AMP4SS platform.

The A Coruña project consisted of 34 Bluetooth detectors, implemented in the city as shown in Figure 6. Figure 6a shows the area covered by the system, while Figure 6b presents the placement of the sensors in the network.

**Figure 7:**
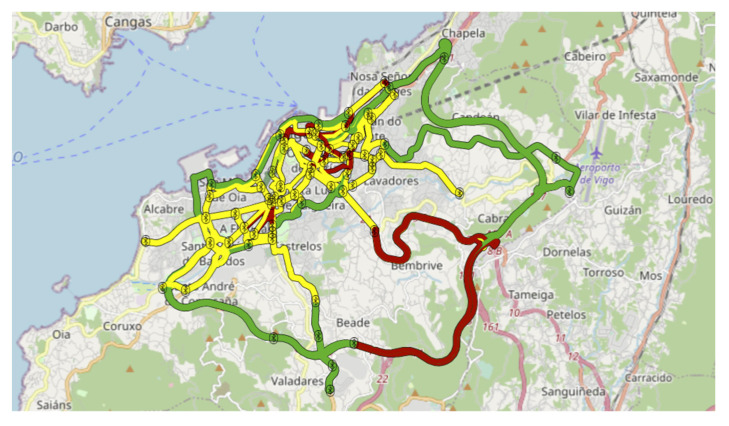
Vigo implementation (from VCC platform).

The Vigo project consisted of 69 Bluetooth detectors, implemented in the city as shown in Figure 7.

**Figure 8:**
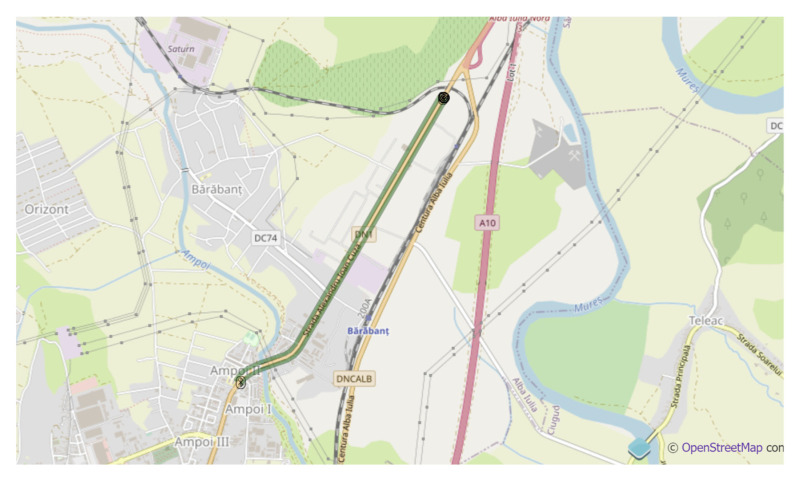
Alba Iulia implementation (from VCC platform).

The Alba Iulia project consisted only of 2 Bluetooth detectors, implemented in the city as shown in Figure 8. Despite the reduced number of detectors, the Alba Iulia project provided all the necessary data for a Bluetooth–RADAR comparison: being placed along a route, with only insignificant road junctions between them, it was very easy to compare the information provided by Bluetooth detectors with the RADAR data, as the latter sensor was placed on the same road section. This comparison will be presented below.

It may be noted that for each project a combination of Bluetooth detectors was implemented that could identify the directions of movements for the vehicles, supplemented with RADAR detectors that could count, in certain points, the exact number of vehicles passing, validating and calibrating the Bluetooth data. Also, this comparison was useful to determine the percentage of vehicles detected by the Bluetooth solution from the total number of vehicles detected on the street. 

## 4. Bluetooth Detection Information Analysis

### 4.1. Data Provided by the System, via Dedicated Platforms

**Figure 9:**
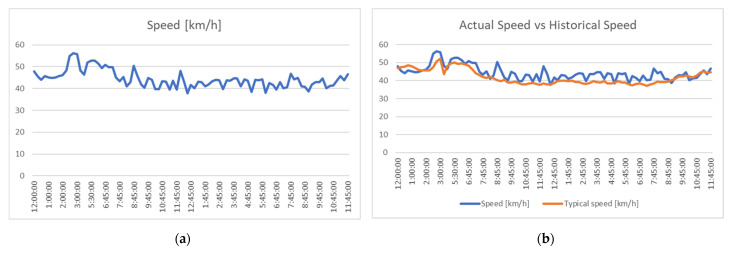
Speed information: (**a**) determined from probes; (**b**) determined from probes compared to typical values.

**Figure 10:**
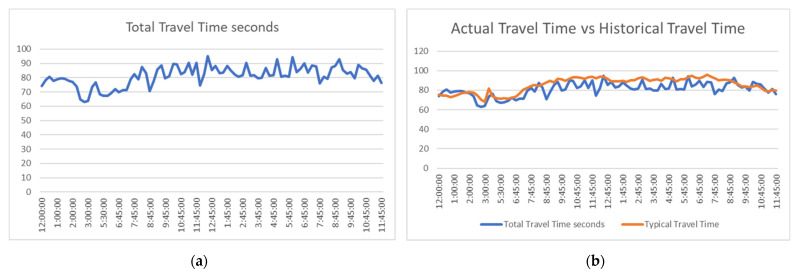
Travel time information: (**a**) determined from probes; (**b**) determined from probes compared to typical values.

Both platforms can visualize real time and historical data, including vehicle speeds, travel time and congestion levels. As an example, the next figures are represented the results obtained with the system for a 24 h period of time on the day of 24 August (Tuesday): in Figure 9, vehicle speeds were identified in real time, based on detected probes, and the speed was compared to historic data (as the system records data over large periods of time), in Figure 10, the travel time on each section of the road network is presented and the current data were compared to historic data. The typical speed and the typical travel time are based on the median of the last 26 measurements of this exact same weekday and time, and the values are recalculated Sunday night, every week.

We must emphasize that the data are not relevant per se, but are included for the reader to better understand the capabilities of the system that was tested.

**Figure 11:**
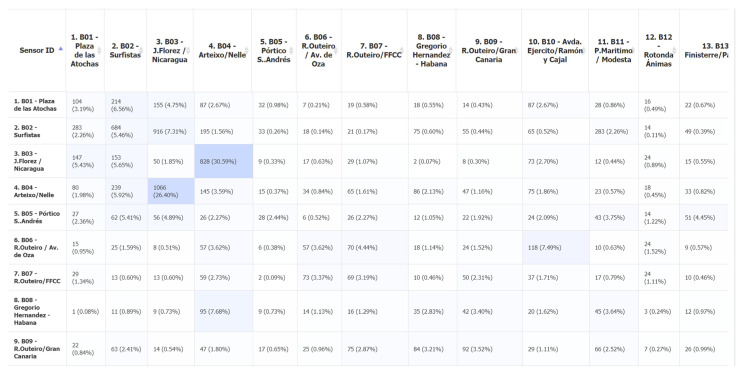
O-D matrix (example from VCC platform).

Considering the way this system functions by matching data between sensors, it results a unique capability of generating origin–destination (O-D) matrices, valuable data for traffic studies and mobility plans that no other detector beside very expensive dedicated CCTV systems with license plate recognition systems can provide. Having in mind that data for O-D are usually collected manually, and also as a percentage of the total trips, this system proves to be a viable alternative for traffic information collection. In Figure 11, a portion of the O-D matrix generated for A Coruña project is presented.

### 4.2. Data Reliability

This system presents many features, but it is important to be proven reliable compared to existing solutions. Bluetooth is still a new technology for implementation in vehicles and older vehicles without this technology will not be detected unless Bluetooth devices are in use. From the information provided by TrafficNow, the installed systems have detected many device descriptions like “TomTom” and “Parrot hands-free,” proving that even older cars can be identified using this solution.

**Figure 12:**
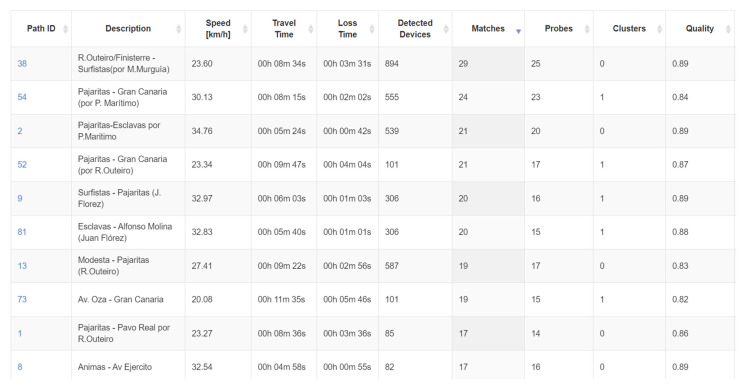
Data from VCC platform—matches vs. devices detected.

**Figure 13:**
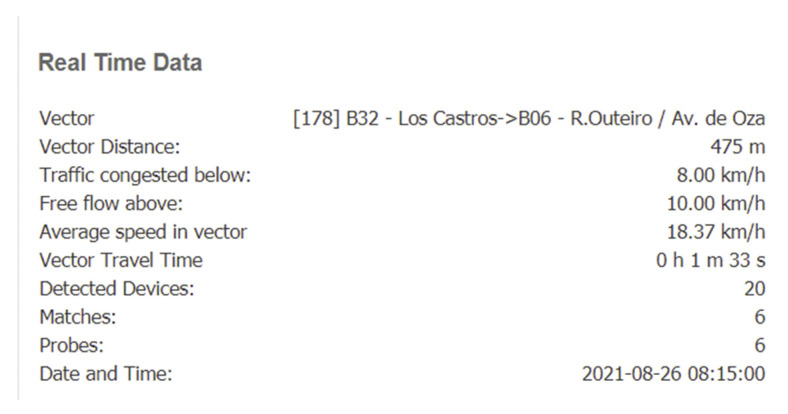
Data from VCC platform—details that can be obtained for each detection vector.

To evaluate the system, data were extracted from VCC for several projects and will be presented below. As a general overview, the information that available in the platform is presented in Figure 12 and Figure 13.

Figure 12 presents the data for each connection between two devices: one can see the average speed that was computed for the link, current travel time and, compared to typical data, loss time calculated for the current situation, number of detected devices, matches and probes (obtained from one- or two-level data filtering), clusters (groups of devices travelling in the same pattern), and an internal estimation of the service quality.

Figure 13 is presents the information obtained via a click on a specific link. One can see the distance between sensors and the thresholds used to color the streets, including: speed limits that determine congestion/free flow, average historical speed, travel time, and information related to device detection (detected devices/matches/probes).

The analysis continued by extracting data for several days and trying to determine a match success rate from the total devices that were identified by Bluetooth detectors. This rate reveals the algorithm’s “effort” to filter data and provide information directly related to vehicles in the road network. For each project, we present a comparison between matches and total detected devices. Then we calculated the percentage of the detected devices that were found as matches between sensors, and then grouped the values obtained, to frame the results in intervals.

**Figure 14:**
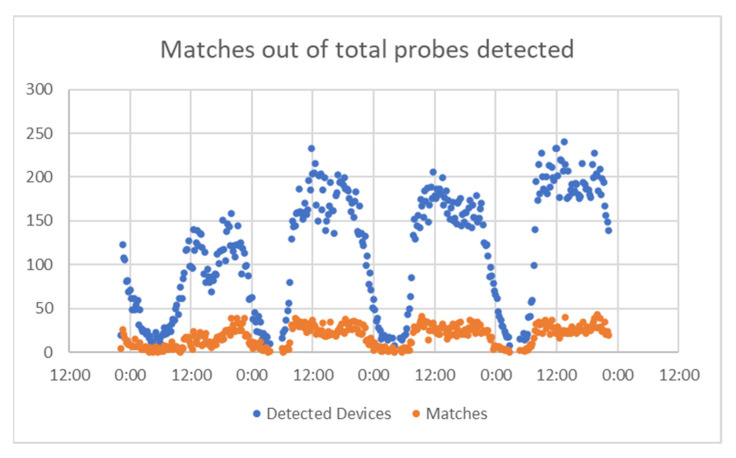
Matches vs. total probes, A Coruña.

**Figure 15:**
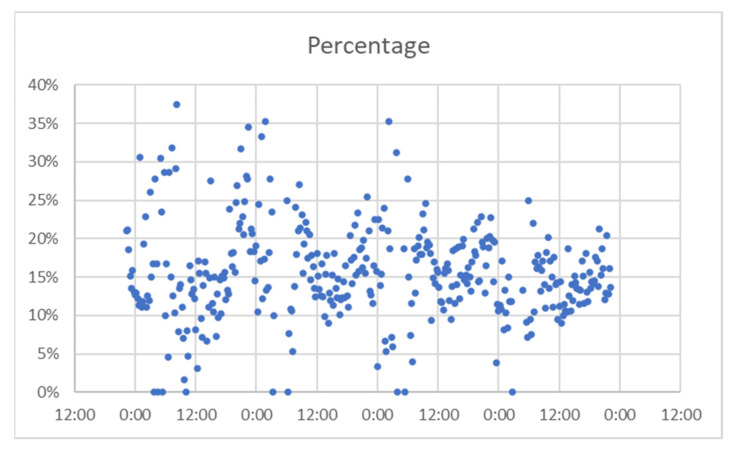
Percentage of successful detection, A Coruña.

**Figure 16:**
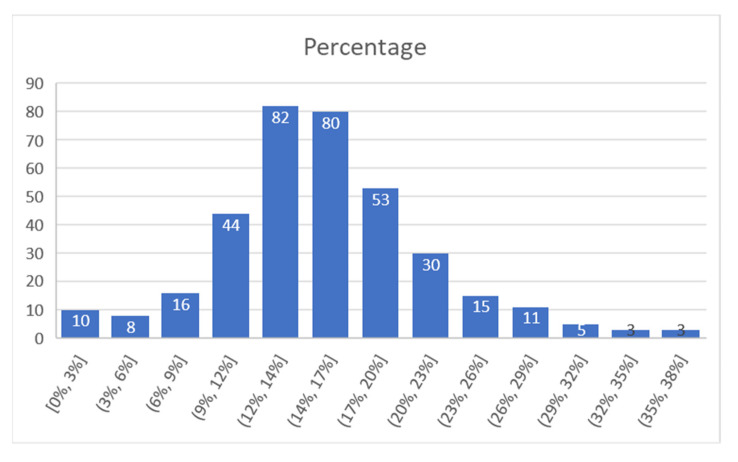
Percentage of successful detection (intervals), A Coruña.

For A Coruña, considering the time interval of 22–25 August, Figure 14 presents the number of matches from the total detected devices. This was used to check if there was the same pattern for both, or there were significant differences. In Figure 15 represent the percentage of the detected devices that were considered matches; this seems to be spread in a wide range, so it resulted the need to group them in narrower intervals. This is shown in Figure 16.

For the A Coruña, project a total of 360 values were gathered in the 4-day period of measurement time considered. From these, it resulted that 82 values were in a detection interval of 12–14%, and 80 values were in a detection interval of 14–17%. Therefore, 45% of the measurements were in the 12–17% match interval. 

The average match value obtained for the whole interval was 16%, while the median was 15%.

**Figure 17:**
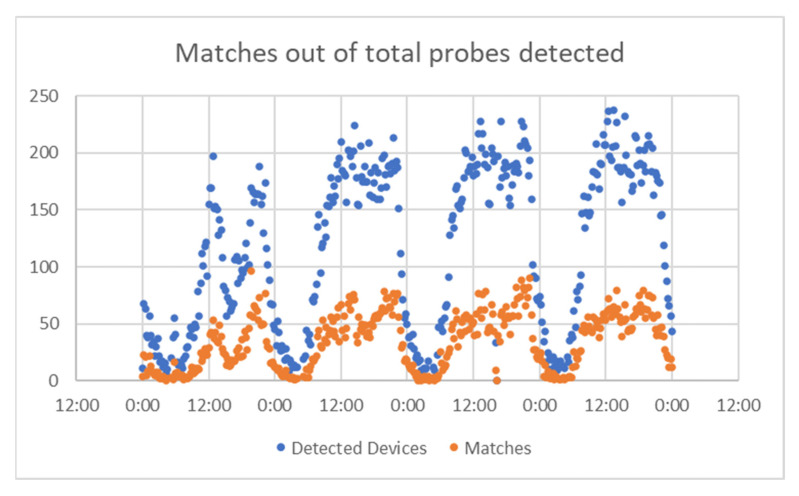
Matches vs. total probes, Vigo.

**Figure 18:**
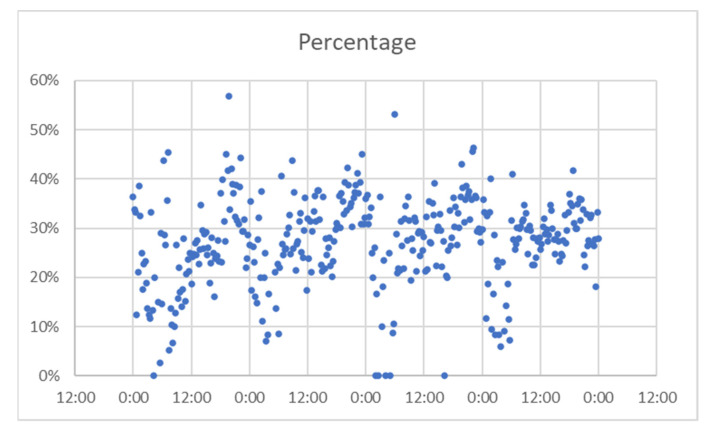
Percentage of successful detection, Vigo.

**Figure 19:**
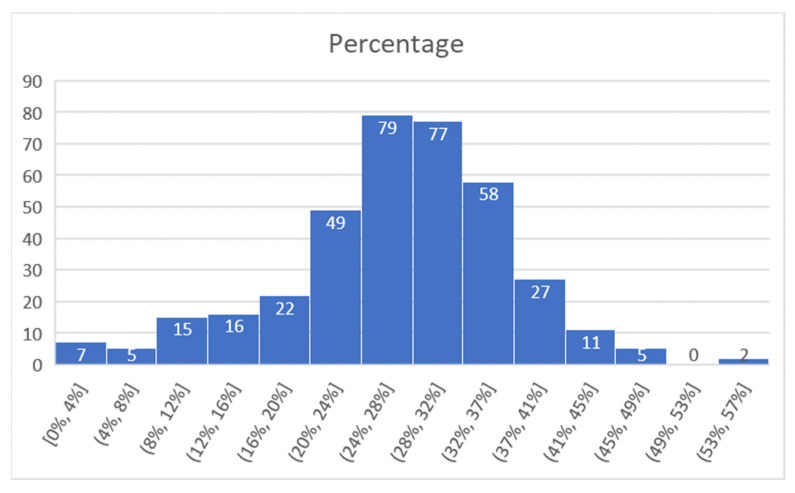
Percentage of successful detection (intervals), Vigo.

For Vigo, considering the time interval of 22–25 August, the data obtained are presented in Figure 17 (number of matches from the total detected devices), Figure 18 (percentage of the detected devices that were considered matches), and Figure 19 (grouping of percentage data into intervals).

For the Vigo project, a total of 360 values were gathered in the 4-day period of measurement time considered. From these, it resulted that 79 values were in a detection interval of 24–28%, and 77 values were in a detection interval of 28–32%. Therefore, 56% of the measurements were in the 24–32% match interval. 

The average match value obtained for the whole interval was 27%, while the median was 28%.

**Figure 20:**
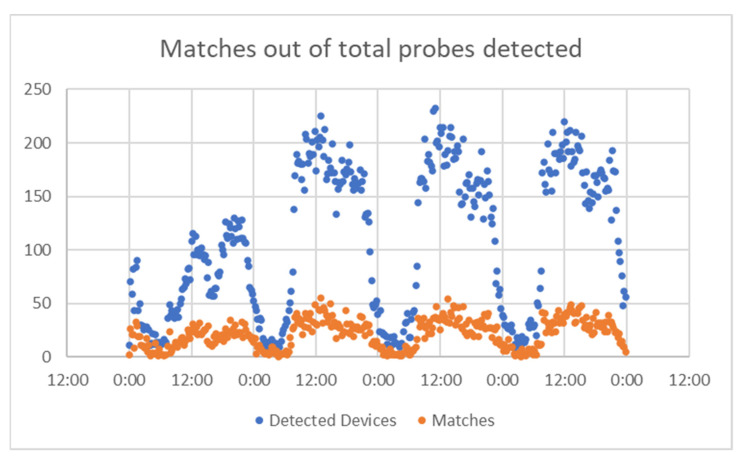
Matches vs. total probes, Alba Iulia.

**Figure 21:**
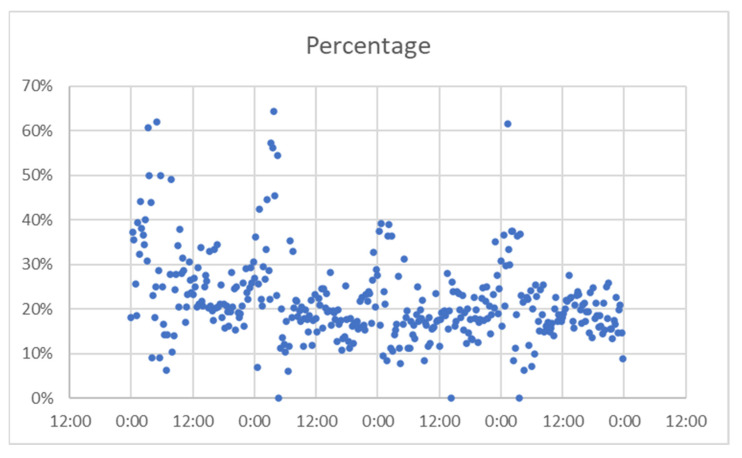
Percentage of successful detection, Alba Iulia.

**Figure 22:**
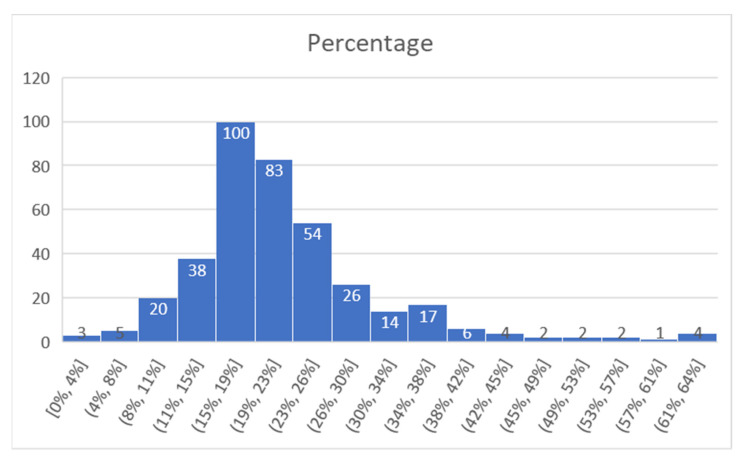
Percentage of successful detection (intervals), Alba Iulia.

For Alba Iulia, the time interval chosen was 25–28 August 2019 (the data collection project ended there), and data obtained are presented in Figure 20 (number of matches from the total detected devices), Figure 21 (percentage of the detected devices that were considered matches), and Figure 22 (grouping of percentage data into intervals).

For the Alba Iulia project, a total of 360 values were gathered in the 4-day period of measurement time considered. From these, it resulted that 100 values were in a detection interval of 15–19%, and 83 values were in a detection interval of 19–23%. Therefore, 51% of the measurements were in the 15–23% match interval. 

The average match value obtained for the whole interval was 22%, while the median was 20%.

**Figure 23:**
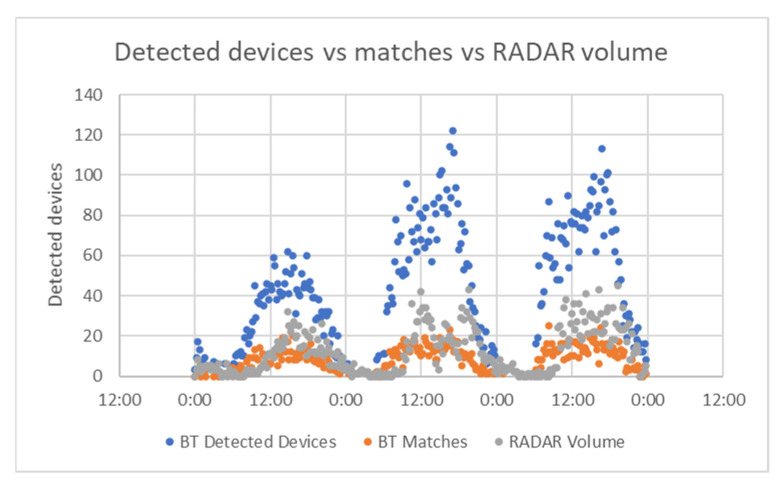
Bluetooth detected devices vs. Bluetooth matches vs. RADAR volume.

For the Alba Iulia project, as the data were recorded in the database at the same time for both Bluetooth and RADAR, we were able to make a comparison between the two technologies to determine if Bluetooth was a suitable solution for traffic detection. First, we tried to represent, on the same chart, all the devices detected by Bluetooth, the matches that were considered as proper traffic data, and the volume of vehicles detected by RADAR in the same area. The datasets were split into 15 min intervals, and the detected matches via Bluetooth were compared with the RADAR results. The values are presented in Figure 23.

**Figure 24:**
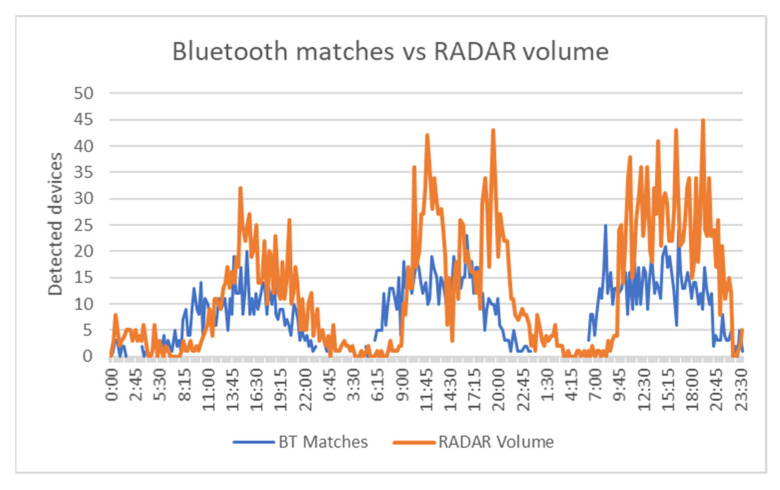
Bluetooth matches vs. RADAR volume—3-day period.

The chart indicates that the Bluetooth detectors are able to identify more objects than vehicles on the street (this was expected considering the wide spreading of Bluetooth technology in wearables and other devices). To better view the Bluetooth matches (the object filtered as vehicles) compared to actual vehicles on the street identified via RADAR technology, Figure 24 was drawn. On the X axis is the number of data points, and on the Y axis is the number of devices.

**Figure 25:**
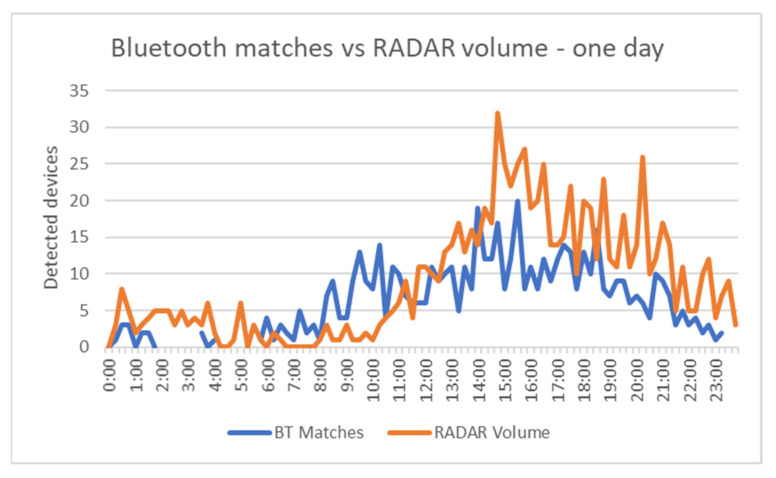
Bluetooth matches vs. RADAR volume—one-day period.

Figure 24 reveals that the traffic pattern monitored by Bluetooth was almost similar to the one identified via RADAR technology. To better view the details, Figure 25 presents the data for only one day.

Analyzing the total values, there were 3439 vehicles detected by RADAR, and 2131 vehicles identified via Bluetooth (62%).

### 4.3. Discussions

The results presented above prove that Bluetooth detectors are suitable to be used as a cheaper alternative to classic vehicle detectors. First of all, the systems used were able to properly filter the detected devices; the percentage of matches out of the total detected devices was relatively low in all three use cases, proving the efficacity of the system to filter vehicles from all the detected objects. This can be seen by comparing the Bluetooth matches and the number of vehicles detected by RADAR, which followed the same pattern during the test period, revealing the similarity of the information provided by this system to the real traffic behavior. Regarding the Bluetooth matches, the resulted overall detection rate of 62% proved to be higher than the values obtained in past studies. For example, in [5,24], when the authors also used RADAR as the ground truth, the detection rate was between 5.39% and 12.19%. Although the detection rate is relatively high, it is still difficult to replace classic detectors with Bluetooth ones. As seen in Figure 25, Bluetooth matches are usually lower than RADAR volume, considering the lack of Bluetooth devices in some vehicles, but there are times when the number of matches is higher, suggesting that better filtering is probably required. The increase in the detection rate is still valuable, as it can improve the confidence in other traffic parameters and their results obtained with the help of Bluetooth detection systems.

Secondly, the comparison between Bluetooth matches and RADAR detections confirmed what other studies discovered, that there is a strong correlation between the two data sources, and that performing a calibration could be a solution for correcting missing information due to the absence of Bluetooth technology in some vehicles. Still, although this technology is unable to provide a correct vehicle count at this moment, Bluetooth data can be successfully used to derive other traffic information with high accuracy, compared to other types of detectors, including traffic speed and density, travel time, or O-D matrices. Since other traffic parameters can be obtained in addition to the vehicle count itself (which is easily detected by all classic traffic detectors), they should be further validated by other technologies, like RADAR (for traffic speed), or video cameras (for travel time and O-D matrices) to properly determine the possibility of replacing those technologies, or complementing them with Bluetooth detectors to improve their accuracy. This was not the main purpose of this paper and requires more specific information collection (like vehicles’ licence plates identification) to be able to determine travel times or O-D pairs, and will be the scope of our future work.

## 5. Conclusions

As a conclusion, a great amount of data can be collected via Bluetooth detectors, therefore an appropriate data filtering and analysis method is essential to separate vehicles from other objects in the road network. The detection rate obtained was good, but it is obvious that, at this moment, Bluetooth technology must be complemented with classic detectors in certain points, to validate the data and calibrate the results. Also, from the data gathered in different cities, preliminary field implementation tests are necessary to determine the appropriate data collection points and the requirements of classic detectors to calibrate the data. However, the Bluetooth-RADAR comparison revealed that both follow the same traffic patterns, proving the relevance of this traffic detection solution.

In the end, we must emphasize the advantage of this technology is that it is inexpensive and thus it sometimes more economic sense than classic sensors densely placed in the city. Also, we must consider the types of information that one can get: in addition to the number of vehicles (detected with a certain precision), Bluetooth detection is capable of generating origin–destination matrices (valuable data for traffic studies and mobility plans), that no other detector (beside expensive CCTV systems with license plate recognitions systems) can provide, making it a useful traffic data collection system.

For future work, we consider that new tests are constantly needed due to the evolution of vehicle technologies. Each parameter provided by such a system (traffic count (currently inaccurate), speeds, travel times, etc.) should be further studied, and specific methodologies must be developed to validate each of them using other relevant traffic information sources.

## Figures and Tables

**Table 1:** Bluetooth potential applications [15].

S/N	Application	Traffic Metric	Benefit
1	Link-flow estimation for congestion control	Link-Flow	Cost benefit, improved traffic prediction, optimised road through congestion management
2	Data augmentation	Link-Flow/Journey time/Speed	Improved accuracy, avoidance of network failure and better reliability
3	Temporal and spatial status network monitoring	O-D matrix/Journey time/Speed	Enhanced traffic management leading to safety, cost and health benefits
4	Support for network optimisation	O-D matrix/Journey time/Speed	Enhanced traffic management leading to safety, cost and health benefits, optimised road network
5	Traffic impact analysis	O-D matrix	Health and cost benefits as well as social and psychological benefits
6	Incident detection	Journey time/speed	Enhanced traffic management through rapid response to emergency situations
7	Dwell time analysis	Journey time	Cost and safety benefits, enhanced fleet management and vehicle monitoring
8	Travel time index study	Journey time	Cost benefit, variability index and congestion management for an optimised road
9	Speed limit compliant level monitoring	Journey speed	Safety benefit
10	Level of service analysis	Flow/Speed	Enhanced traffic management
11	Density estimation	Flow/Speed	Enhanced traffic management
12	Decision support system	O-D matrix/Journey time/Speed	Enhanced traffic management

## Data Availability

Restrictions apply to the availability of these data. Data was obtained from TrafficNow, Spain, and Analytiqum Innovative Solutions, Romania, and are available from the corresponding author with the permission of TrafficNow, Spain, and Analytiqum Innovative Solutions, Romania.
